# Endoscopy biopsy is not efficiency enough for diagnosis of mucinous colorectal adenocarcinoma

**DOI:** 10.1007/s12672-021-00443-4

**Published:** 2021-10-25

**Authors:** Shuai Xiao, Jia Huang, Yiwei Zhang, Rong Tang, Yunhua Xu, Rongfang He, Qiulin Huang, Jun Ouyang, Xuyu Zu, Xiuda Peng, Kai Fu

**Affiliations:** 1 The First Affiliated Hospital, Institute of Oncology, Hengyang Medical School, University of South China, Hengyang, 421001 Hunan China; 2 The First Affiliated Hospital, Department of Gastrointestinal Surgery, Hengyang Medical School, University of South China, Hengyang, 421001 Hunan China; 3 The First Affiliated Hospital, Department of Pathology, Hengyang Medical School, University of South China, Hengyang, 421001 Hunan China; 4 The Second Affiliated Hospital, Department of Surgery, Hengyang Medical School, University of South China, Hengyang, 421001 Hunan China; 5 Institute of Molecular Precision Medicine and Hunan Key Laboratory of Molecular Precision Medicine, Xiangya Hospital, Central South University, Changsha, 410008 Hunan China

**Keywords:** Colorectal cancer, Mucinous adenocarcinoma, Endoscopy biopsy, Pathological diagnosis

## Abstract

**Background:**

Endoscopy biopsy (EB) is the standard diagnostic method for colorectal cancer (CRC), whereas its accuracy and efficiency in mucinous adenocarcinoma (MAC) initial diagnosis is unclear.

**Methods:**

The initial EB and postoperative specimen (PS) pathological diagnosis of MAC from two centers were retrospectively collected and analyzed. The accuracy and efficiency of initial EB compared with PS pathological diagnosis were analyzed. The potential factors which would affect the initial EB diagnosis of MAC were analyzed.

**Results:**

280 and 78 eligible cases were enrolled in this study from two centers respectively. The initial EB diagnosis accuracy for MAC were 84.62% and 83.33%. However, among the cases of PS diagnosis with MAC, the diagnostic efficiency of initial EB was only 36.49% and 32.50% respectively. Lower tumor differentiation and more EB number were associated with an increased probability for the EB diagnosis of MAC, but only tumor differentiation was an independent diagnositic factor for EB in the two cohorts.

**Conclusions:**

The accuracy of initial EB with MAC is high, but the diagnostic efficiency was extremely low. Tumor differentiation and EB number were associated with the diagnosis efficiency of MAC before surgery.

**Supplementary Information:**

The online version contains supplementary material available at 10.1007/s12672-021-00443-4.

## Introduction

Colorectal cancer (CRC) is one of the most prevalent and lethal cancer and accounts for about 10% of all newly diagnosed and deaths of cancer in the world [1, 2]. Histology is the key for diagnosis and treatment guidance of cancer. The most common pathological subtype is non-specific adenocarcinoma (AC) which accounts for about 85% of all CRC patients, mucinous adenocarcinoma (MAC) is the second leading subtype with about 10%-20% proportion of total CRCs[3, 4]. MAC is defined by the characteristic of at least 50% of the tumor volume is comprised of abundant mucinous components [3]. The clinicopathological features and prognosis are distinct, although both MAC and AC belong to adenocarcinoma of CRC. MAC patients always had advanced tumor stage, poor chemotherapy response and poor long-term survival compared with AC patients, which indicated more attention should be paid to diagnosis and treatment of MAC [3–8]. Therefore, correctly diagnosis of MAC before treatment has great value to guide clinical management and improve prognosis, especially for which need neoadjuvant therapy.

Endoscopy with biopsy is the “gold standard” for the preoperative diagnosis of CRC with extremely high accuracy and guiding significance for treatment [2]. Endoscopy could observe the whole large intestine and tumor gross morphology, as well as obtain biopsy specimens. However, the diagnostic role of endoscopy in MAC of CRC is still unknown. A few studies from western countries showed that the accuracy of endoscopy biopsy in the diagnosis of MAC was even less than 20%, which was far lower than the average diagnosis rate of CRC [9, 10]. Therefore, additional research is needed to evaluate the real role of endoscopy biopsy in MAC diagnosis.

## Materials and methods

### Study design

We performed a retrospective study involving patients who underwent both endoscopy and surgery for CRC from the same hospital between January 2010 and January 2019, from Xiangya Hospital of Central South University (Changsha cohort) and from the First Affiliated Hospital of University of South China (Hengyang cohort). All of consecutive cases had been reported as MAC or mixed MAC (AC mixed with MAC, which the proportion of MAC is less than 50%) by endoscopic biopsy and/or postoperative specimen pathological diagnosis, and the information were recorded including endoscopy results and pathological information of postoperative specimens. Patients accepted a low-residue fluid diet 1 day before colonoscopy, and orally took 4 bags of polyethylene glycol-electrolyte powder with 2000–3000 mL of warm water. The colonoscopy was performed by standard endoscope (EC-590WM, Fujinon Corp., Omiya, Japan) about 4 h after intestinal cleansing. Biopsy forceps (ATE-QYQ-C-23 × 1800, AteTec™, Jiangsu, China), which was about 6 mm in length when open, were used to take the intestinal suspicious tumors, then the tissues were formalin fixed and sent for pathological examination. If the surface of the lesion is out of flatness, biopsy should be performed from the sunken and the protuberant lesion. The colonoscopy quality indicators were accord with the recommendations of the ASGE guideline [11]. The surgery for CRCs was in accordance with CME or TME depending on the location of the tumor [2].

The "gold standard" for MAC diagnosis was postoperative specimen pathological diagnosis. The primary endpoint of this study was the diagnosis of MAC by endoscopic biopsy, the secondary endpoint were the diagnosis of MAC by postoperative specimen and failure diagnosis of MAC by biopsy. The postoperative specimen MAC cases were enrolled for potential diagnostic influencing factors analysis. Due to the diagnosis of MAC needs at least 50% of the tumor volume is comprised of mucinous components, which makes biopsy specimens potentially hard to fully reflect the diagnosis of MAC due to the small size and fragility of the tissues, MAC and mixed MAC cases by endoscopic biopsy were considered as the initial diagnosis of MAC.

This study was approved by the ethics committee of Xiangya Hospital of Central South University and the First Affiliated Hospital of University of South China and was in accordance with the Declaration of Helsinki.

### Inclusion criteria, exclusion criteria and research factors

Inclusion criteria: 1. all of patients had undergone both endoscopy biopsy and surgery in the same hospital; 2. MAC or mixed MAC were diagnosed by biopsy and/or postoperative specimen pathological diagnosis; 3. the time interval from the biopsy to surgery was no more than 1 month; 4. the pathological diagnosis for MAC meet the World Health Organization (WHO) classification criteria [3]. The exclusion criteria were: 1. patients had not performed endoscopy biopsy or surgery in the same hospital; 2. the time interval from biopsy to surgery was more than 1 month; 3. patients didn’t obtain pathological confirmation of MAC or mixed MAC either on biopsy or postoperative specimen; 4. colorectal metastatic lesion; 5. patients with chemotherapy or radiotherapy before biopsy and/or surgery; 6. the biopsy number of endoscopy was unknown. The pathological diagnosis of MAC was evaluated by two independent pathologists. MAC is defined as > 50% of the tumor is composed of extracellular mucin that contain overt malignant epithelium [3]. Mixed MAC is defined as less than 50% of the tumor is composed of extracellular mucin, and most of the tumor is constituted with the adenocarcinoma NOS.

The research factors were collected and analyzed in this study including the baseline data such as sex, age, tumor location, tumor size, gross type, differentiation, pT and pN stage, biopsy number and polyps’ status.

### Statistical analysis

All statistical analyses were performed using SPSS version 22.0 (SPSS Inc., Chicago, IL, USA). The categorical variables expressed as a percentage (%), the continuous variables used the mean ± standard deviation, the categorical variables between groups were compared using the chi-square test, the continuous variables were compared using One-Way ANOVA. The factors impacting the diagnostic accuracy of endoscopy were analysed by univariable and multivariable binary logistic regression analysis, the covariates of which P-value less than 0.20 were included in multivariable regression analyses. A P-value < 0.05 was considered statistically significant.

## Results

### The general characteristics of MAC patients

**Fig. 1:**
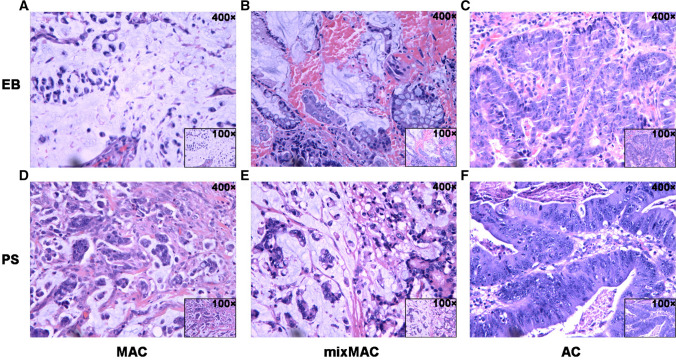
The representative hematoxylin–eosin (HE) staining images of the histology subtypes of endoscopy biopsy and postoperative specimen. **A** Mucinous adenocarcinoma (MAC), **B** mixed MAC (mixMAC) and **C** non-specific adenocarcinoma (AC) from endoscopy biopsy; **D** MAC, **E** mixMAC and **F** AC from postoperative specimen

A total of 280 eligible patients diagnosed with MAC or mixed MAC by endoscopy biopsy (EB) and/or postoperative specimen (PS) pathological examination were included in Changsha cohort, including 158 males and 122 females. The age range was 24–91 years, with a median age of 54 years. There were 124 cases with tumors located at colon proximal to the splenic flexure, 64 cases distal to the splenic flexure and 92 cases in rectum. Other detailed clinical and pathological information were provided in Table 1. In this study, all cases were confirmed as CRC by postoperative specimen pathological examination. Among them, 262 cases were confirmed as CRC by preoperative biopsy, and the diagnostic accuracy of CRC by preoperative biopsy was 93.57% (262/280) (Table 1). The representative images of MAC, mixed MAC and AC from EB or PS were showed in Fig. 1.

**Table 1:** The baseline characteristics of colorectal MAC patients from Changsha cohort

Variables	Cases (n)	Variables	Cases (n)
Age (year)	55.0 ± 12.82	Gross type	
≤ 60	182	Mass	100
> 60	98	Ulcer	151
Location		Unknown	29
Proximal	124	PS with MAC	
Distal	64	Absent	4
Rectal	92	Present	276
EB with MAC		PS definition	
Absent	240	AC	4
Present	40	MAC	74
EB definition		mixMAC	202
AC	222	PS differentiation	
MAC	26	Well/moderate	196
mixMAC	14	Poor/undifferentiation	68
Non-Cancer	18	Unknown	16
EB differentiation		PS size (CM)	5.53 ± 2.49
Well/moderate	196	≤ 5	141
Poor/undifferentiation	57	> 5	122
Unknown	27	Unknown	17
EB number	3.17 ± 1.53	PS pT	
≤ 2	108	1–2	65
> 2	172	3–4	215
Polyps status		PS pN	
Absent	237	N0	152
Present	43	N1	66
Sex		N2	62
Male	158		
Female	122		

EB, endoscopic biopsy; PS, postoperative specimen; AC, non-specific adenocarcinoma; MAC, mucinous adenocarcinoma; mixMAC, mixed MAC; pT, pathological tumor staging; pN, pathological nodal staging

Consistent with the results above, there were 78 patients diagnosed with MAC or mixed MAC by postoperative specimen pathological examination from Hengyang cohort, and the diagnostic accuracy of CRC by preoperative biopsy was 96.15% (75/78), the detailed information was showed in Supplementary Table S1.

### Accuracy of initial endoscopy biopsy for MAC

In the Changsha cohort, the initial EB of pathological diagnosis was confirmed in 26 patients with MAC (E-MAC) and 14 patients with mixed MAC (E-mixMAC). The PS pathological diagnosis was confirmed in 74 with MAC (P-MAC) and 202 cases with mixed MAC (P-mixMAC). The accuracy of P-MAC patients detected by E-MAC was 84.62% (22/26), while the accuracy declined to 67.50% (27/40) when detected with E-MAC or E-mixMAC. However, the accuracy of E-mixMAC to P-mixMAC was only 42.86% (6/14) (Table 2). These findings were also confirmed by the Hengyang cohort, that the accuracy of P-MAC patients detected by E-MAC was 83.33% (5/6), while the accuracy declined to 76.50% (13/17) when detected with E-MAC or E-mixMAC, and the accuracy of E-mixMAC to P-mixMAC was only 27.27% (3/11).

**Table 2:** The crosslinking table of endoscopic biopsy and postoperative specimen diagnosis of MAC

	EB				Total
	AC	MAC	mixMAC	Non-Cancer	
PS					
AC	0	1	3	0	4
MAC	39	22	5	8	74
mixMAC	183	3	6	10	202
Total	222	26	14	18	280

### Efficiency of initial endoscopy biopsy for MAC

Then, we analyzed the diagnostic efficiency of initial endoscopy biopsy for P-MAC patients. The results showed that the P-MAC patients, a total of 36.49% (27/74), were identified by E-MAC (22/74, 29.73%) or E-mixMAC (5/74, 6.76%) in initial endoscopy biopsy cases. The P-mixMAC patients were identified by E-mixMAC in only 2.97% (6/202) cases of initial endoscopy biopsy (Table 2). The results were also confirmed by Hengyang cohort that the diagnostic efficiency for P-MAC or E-mixMAC, a total of 32.50% (13/40), was only 12.50% (5/40) and 20.00% (8/40) in initial endoscopy biopsy cases. The P-mixMAC patients were identified by E-mixMAC in only 7.89% (3/38) cases of initial endoscopy biopsy (Supplementary Table S2).

### Factors impacting the diagnosis of MAC by initial endoscopy biopsy

**Fig. 2:**
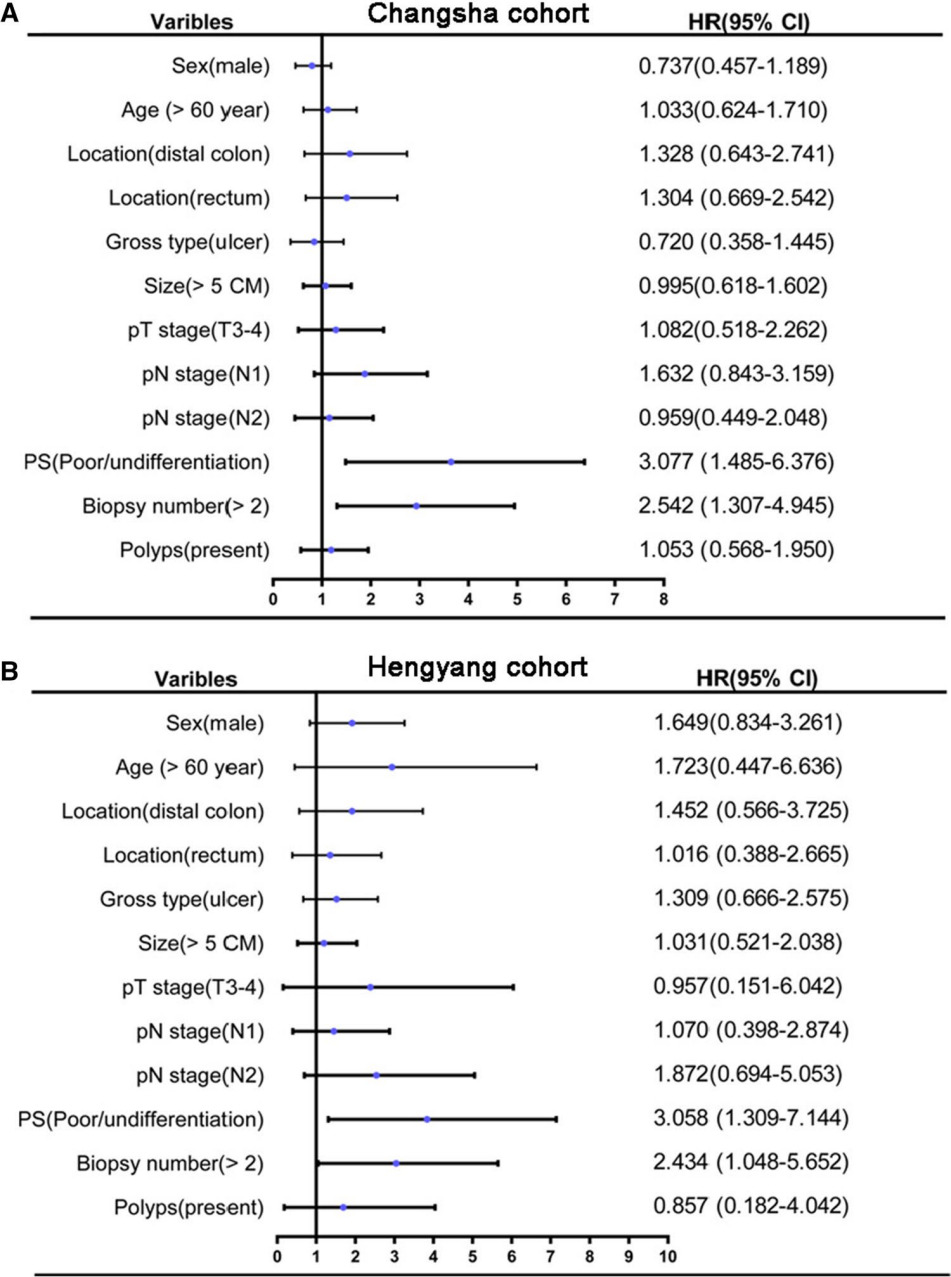
The forest plots of the influencing factors for the diagnosis of MAC with endoscopy biopsy. **A** The Changsha cohort showed lower tumor differentiationand more EB numberwere associated with an increased diagnosis probability; **B** the Hengyang cohort showed the similar results

Since patients diagnosed with MAC after surgery had low preoperative endoscopy biopsy diagnosis efficiency, the potential influencing factors were retrospectively analyzed by logistic regression. Accordingly, the 74 P-MAC cases were divided into E-MAC positive group and E-MAC negative group. Results showed the age, sex, tumor location, gross type, postoperative tumor size, pT, pN stage and polyp status of patients had no significant difference between E-MAC positive and E-MAC negative groups (all P > 0.05, respectively, Table 3), but the biopsy number and tumor differentiation of postoperative specimen between the two groups had dramatic differences (both P < 0.05, Table 3). Further binary logistic regression showed biopsy number > 2 (RR = 2.542, CI 1.307–4.945) and tumor poor/undifferentiation (RR = 3.077, CI 1.485–6.376) were associated with an increased probability for the diagnosis of E-MAC with endoscopy biopsy (Table 4, Fig. 2A). The multivariable analysis also confirmed biopsy number > 2 (RR = 2.570, CI 1.248–5.295) and poor/undifferentiation (RR = 3.028, CI 1.353–6.777) were independent factors for impacting the diagnosis of E-MAC with endoscopy biopsy (Table 4).

**Table 3:** The clinicopathological features of colorectal MAC patients according to the endoscopic biopsy results

Variables	E-MAC positive (27 cases)	E-MAC negative (47 cases)	P value
Sex			
Female	15	19	
Male	12	28	0.209
Age (year)			
≤ 60	18	32	
> 60	9	15	0.900
Location			
Proximal	8	23	
Distal	8	10	
Rectal	11	14	0.269
Gross type			
Mass	7	18	
Ulcer	14	26	
Unknown	6	3	0.122
PS size (CM)			
≤ 5	15	26	
> 5	12	21	0.984
PS pT stage			
1–2	3	6	
3–4	24	41	0.833
PS pN stage			
0	8	22	
1	6	11	
2	13	14	0.241
PS differentiation			
Well/moderate	6	29	
Poor/undifferentiation	15	8	
Unknown	6	10	0.001
EB number			
≤ 2	3	21	
> 2	24	26	0.002
Polyps status			
Absent	22	39	
Present	5	8	0.871

E-MAC, MAC diagnosed with endoscopy biopsy

**Table 4:** Logistics analyses for the risk factors affecting the endoscopic biopsy diagnosis of colorectal MAC

Variables	Univariate analysis		Multivariable analysis	
	RR (95% CI)	P value	RR (95% CI)	P value
Sex				
Female	1	0.211		
Male	0.737 (0.457–1.189)			
Age (year)				
≤ 60	1	0.900		
> 60	1.033 (0.624–1.710)			
Location				
Proximal	1	0.276		
Distal	1.328 (0.643–2.741)			
Rectal	1.304 (0.669–2.542)			
Gross type				
Mass	1	0.141	1	0.305
Ulcer	0.720 (0.358–1.445)		0.810 (0.354–1.853)	
PS size (CM)				
≤ 5	1	0.984		
> 5	0.995 (0.618–1.602)			
PS pT stage				
1–2	1	0.834		
3–4	1.082 (0.518–2.262)			
PS pN stage				
0	1	0.249		
1	1.632 (0.843–3.159)			
2	0.959 (0.449–2.048)			
PS differentiation				
Well/moderate	1	0.003	1	0.016
Poor/undifferentiation	3.077 (1.485–6.376)		3.028 (1.353–6.777)	
EB number				
≤ 2	1	0.006	1	0.010
> 2	2.542 (1.307–4.945)		2.570 (1.248–5.295)	
Polyps status				
Absent	1	0.871		
Present	1.053 (0.568–1.950)			

RR, relative ratio

Accordingly, the Hengyang cohort also found the biopsy number > 2 and tumor differentiation of postoperative specimen between the two groups were significant differences (both P < 0.05, Supplementary Table S3), as well as biopsy number > 2 (RR = 2.434, CI 1.048–5.652) and tumor poor/undifferentiation (RR = 3.058, CI 1.309–7.144) were associated with an increased probability for the diagnosis of E-MAC with endoscopy biopsy (Supplementary Table S4). However, the multivariable analysis showed only the tumor poor/undifferentiation (RR = 2.586, CI 1.055–6.342) was associated with an increased probability for E-MAC. The factor of biopsy number > 2 lost significance by a close P value of 0.089 (RR = 2.205, CI 0.888–5.476, Supplementary Table S4, Fig. 2B).

## Discussion

MAC is the second most common pathological subtype of CRC which accounts for 10%–20% of all CRC worldwide. More importantly, MAC is high peritoneal metastasis and relatively resistant to traditional chemo-radiotherapy, and has a potentially poor prognosis [3, 4]. These features indicate that the therapeutic strategies for advanced MAC are significantly different from that of AC, which was identified by previous studies, especially in the cases that need adjuvant chemo-radiotherapy or neoadjuvant therapy [4, 12]. Therefore, it is of great value to identify the MAC pathological subtype of CRC before treatment decision and prognosis prediction.

Endoscopy biopsy is currently the most common and accurate method to diagnose CRC. However, some studies based on small sample sizes indicated that the diagnosis efficiency of MAC by endoscopy biopsy was particularly low, even less than 10%, leading to the majority of MAC being missed before surgery or other treatments [9, 10, 13]. Accordingly, this study designed MAC biopsy diagnosis as the primary endpoint to answer this question. Our study confirmed that E-MAC was identified in only 36.49% and 32.50% of P-MAC cases respectively in two independent cohorts, which was consistent with the previous studies. It suggests that the diagnostic efficiency of endoscopy biopsy for MAC is extremely low, resulting in more than 70%–90% of MAC cases failing to be diagnosed before surgery or systemic treatment, which would seriously affect the treatment decisions and prognosis assessment. Therefore, there is an urgent need to improve the efficiency of initial endoscopy biopsy of MAC. At the same time, our study of secondary endpoint also found that the MAC samples, which were confirmed by endoscopy biopsy, have a high accuracy with the postoperative specimen pathological diagnosis, 84.62% and 83.33% in the two independent cohorts respectively. These reconfirmed the diagnostic accuracy of endoscopy biopsy is valuably high, and the E-MAC diagnosis is very helpful in deciding treatment strategy [13, 14].

Some potential factors could affect the diagnosis of MAC by endoscopy biopsy, mainly including tumor factors, such as tumor size, gross type, differentiation, staging, etc., and technical factors mainly including the biopsy number and depth [2, 13, 15]. The current study for the secondary endpoint of failure diagnosis of MAC by biopsy found tumor differentiation and endoscopy biopsy number were the impacting factors for MAC initial diagnosis. MAC was initially thought to be poorly differentiated. Whereas later researchers found that MAC could also be well-differentiated, which were consistent with our results [3, 16]. Therefore, the effect of tumor factors on the diagnosis cannot be changed by improving endoscopic procedures, but new methods in assisting initial diagnosis should be explored in the future. The number of endoscopy biopsies is always an important factor affecting the diagnostic accuracy of CRC. In this study we found the diagnostic rate of MAC could be significantly improved with more than 2 biopsy specimens, suggesting that we should pay more attention to endoscopy biopsy for CRC. Other potential reasons for the low diagnostic efficiency of MAC by initial endoscopy may be that the biopsy specimens were brittle, fragmentized, small and shallow which resulted in the pathological diagnosis could not reach the standard of mucous component above 50% of the tumor volume, further affecting the initial biopsy diagnostic efficiency of MAC [17]. In view of this, it is essential to develop new instruments or techniques of large volume or quantitative sampling for endoscopy to improve the biopsy diagnosis of MAC. For example, there were studies that suggested magnetic resonance imaging (MRI) as the differential diagnosis criteria for rectal MAC and AC before treatment with high accuracy reached up to 95% [3, 17].

In conclusion, this study found that MAC diagnosed by initial endoscopic biopsy had considerable accuracy with postoperative pathological diagnosis, but the diagnosis efficiency was extremely low by the comprehensive multi-center retrospective study. Besides, the univariable and multivariable analyses indicated tumor differentiation and biopsy number were the influencing factors for the endoscopic biopsy diagnosis of MAC. Therefore, more attention should be paid to the improvement of biopsy techniques and novel diagnostic methods, such as luorescence probe detection for specific mucin proteins, would increase the efficiency of initial endoscopic diagnosis of MAC in the future.

## Supplementary Information

Additional file 1 (PDF 139 KB)

## Data Availability

The datasets analysed during the current study are not publicly available due the raw data also forms part of an ongoing study, but are available from the corresponding author on reasonable request.
