# Supplementary material for: Endoscopy biopsy is not efficiency enough for diagnosis of mucinous colorectal adenocarcinoma
Source: Discov Oncol. 2021 Oct 25;12:44. doi: 10.1007/s12672-021-00443-4 (PMC8777509; doi:10.1007/s12672-021-00443-4)
Supplement: Supplementary file 1 — Additional file 1 (PDF 139 KB) [file 12672_2021_443_MOESM1_ESM.doc]

**Additional file 1: Table S1** The baseline characteristics of colorectal MAC patients identified by pathological confirmation from endoscopic biopsy or postoperative specimen in Hengyang cohort

| **Variables** | **Cases(n)** | **Variables** | **Cases(n)** |
| --- | --- | --- | --- |
| **Age (year)** | 57.5±11.5 | **Sex** |  |
| ≤ 60 | 40 | Male | 40 |
| ＞ 60 | 38 | Female | 38 |
| **Location** |  | **Gross type** |  |
| Proximal | 31 | Mass | 47 |
| Distal | 20 | Ulcer | 31 |
| Rectal | 27 |  |  |
| **EB with MAC** |  | **PS with MAC** |  |
| Absent | 61 | Absent | 0 |
| Present | 17 | Present | 78 |
| **EB definition** |  | **PS definition** |  |
| AC | 58 | MAC | 40 |
| MAC | 6 | MIX | 38 |
| MIX | 11 |  |  |
| non-Cancer | 3 |  |  |
| **EB differentiation** |  | **PS differentiation** |  |
| Well/moderate | 46 | Well/moderate | 49 |
| Poor/undifferentiation | 19 | Poor/undifferentiation | 29 |
| Unknown | 13 |  |  |
| **EB number** | 3.82±1.51 | **PS size (CM)** | 5.06±2.15 |
| ≤2 | 27 | ≤5 | 51 |
| ＞2 | 51 | ＞5 | 27 |
| **Polyps status** |  | **PS pT** |  |
| Absent | 54 | 1-2 | 15 |
| Present | 24 | 3-4 | 63 |
|  |  | **PS pN** |  |
|  |  | N0 | 40 |
|  |  | N1 | 17 |
|  |  | N2 | 21 |

Abbreviation: EB, endoscopic biopsy; PS, postoperative specimen; AC, non-specific adenocarcinoma; MAC, mucinous adenocarcinoma; mixMAC, mixed MAC; pT, pathological tumor staging; pN, pathological nodal staging.

**Additional file 1: Table S2** The crosslinking table of endoscopic biopsy and postoperative specimen diagnosis of MAC in Hengyang cohort

|  | | **EB** | | | | **Total** |
| --- | --- | --- | --- | --- | --- | --- |
|  |  | **AC** | **MAC** | **mixMAC** | **non-Cancer** |  |
| **PS** | **MAC** | 25 | 5 | 8 | 2 | 40 |
|  | **mixMAC** | 33 | 1 | 3 | 1 | 38 |
| **Total** | | 58 | 6 | 11 | 3 | 78 |

**Additional file 1:Table S3** The clinicopathological features of colorectal MAC patients according to the endoscopic biopsy results in Hengyang cohort

| **Varibles** | **E-MAC positive**  **(13 cases)** | **E-MAC negative**  **(27 cases)** | **P value** |
| --- | --- | --- | --- |
| **Sex** |  |  |  |
| Female | 5 | 17 |  |
| Male | 8 | 10 | 0.145 |
| **Age (year)** |  |  |  |
| ≤ 60 | 5 | 14 |  |
| ＞ 60 | 8 | 13 | 0.427 |
| **Location** |  |  |  |
| Proximal | 4 | 12 |  |
| Distal | 5 | 7 |  |
| Rectal | 4 | 8 | 0.646 |
| **Gross type** |  |  |  |
| Mass | 7 | 18 |  |
| Ulcer | 6 | 9 | 0.433 |
| **PS size (CM)** |  |  |  |
| ≤ 5 | 8 | 17 |  |
| ＞ 5 | 5 | 10 | 0.931 |
| **PS pT stage** |  |  |  |
| 1-2 | 2 | 4 |  |
| 3-4 | 11 | 23 | 0.962 |
| **PS pN stage** |  |  |  |
| 0 | 4 | 15 |  |
| 1 | 4 | 7 |  |
| 2 | 5 | 5 | 0.272 |
| **PS differentiation** |  |  |  |
| Well/moderate | 2 | 17 |  |
| Poor/undifferentiation | 11 | 10 | 0.003 |
| **EB number** |  |  |  |
| ≤ 2 | 2 | 14 |  |
| **＞ 2** | 11 | 13 | 0.022 |
| **Polyps status** |  |  |  |
| Absent | 10 | 20 |  |
| Present | 3 | 7 | 0.845 |

Abbreviation: E-MAC, MAC diagnosed with endoscopy biopsy.

**Additional file 1:Table S4** Logistics analyses for the risk factors affecting the endoscopic biopsy diagnosis of colorectal mucinous carcinoma in Hengyang cohort

| **Varibles** | **Univariate analysis** | |  | **Multivariable analysis** | |
| --- | --- | --- | --- | --- | --- |
|  | **RR(95%CI)** | **P value** |  | **RR(95%CI)** | **P value** |
| **Sex** |  |  |  |  |  |
| Female | 1 | 0.150 |  | 1 | 0.246 |
| Male | 1.649(0.834-3.261) |  |  | 1.415(0.635-3.155) |  |
| **Age (year)** |  |  |  |  |  |
| ≤ 60 | 1 | 0.429 |  |  |  |
| ＞ 60 | 1.723(0.447-6.636) |  |  |  |  |
| **Location** |  |  |  |  |  |
| Proximal | 1 | 0.650 |  |  |  |
| Distal | 1.452 (0.566-3.725) |  |  |  |  |
| Rectal | 1.016 (0.388-2.665) |  |  |  |  |
| **Gross type** |  |  |  |  |  |
| Mass | 1 | 0.435 |  |  |  |
| Ulcer | 1.309 (0.666-2.575) |  |  |  |  |
| **PS size (CM)** |  |  |  |  |  |
| ≤ 5 | 1 | 0.931 |  |  |  |
| ＞ 5 | 1.031(0.521-2.038) |  |  |  |  |
| **PS pT stage** |  |  |  |  |  |
| 1-2 | 1 | 0.962 |  |  |  |
| 3-4 | 0.957(0.151-6.042) |  |  |  |  |
| **PS pN stage** |  |  |  |  |  |
| 0 | 1 | 0.287 |  |  |  |
| 1 | 1.070 (0.398-2.874) |  |  |  |  |
| 2 | 1.872(0.694-5.053) |  |  |  |  |
| **PS differentiation** |  |  |  |  |  |
| Well/moderate | 1 | 0.010 |  | 1 | 0.038 |
| Poor/undifferentiation | 3.058 (1.309-7.144) |  |  | 2.586(1.055-6.342) |  |
| **EB number** |  |  |  |  |  |
| ≤ 2 | 1 | 0.039 |  | 1 | 0.089 |
| ＞ 2 | 2.434 (1.048-5.652) |  |  | 2.205 (0.888-5.476) |  |
| **Polyps status** |  |  |  |  |  |
| Absent | 1 | 0.846 |  |  |  |
| Present | 0.857 (0.182-4.042) |  |  |  |  |

Abbreviation: RR, relative ratio.
